# Managing wetlands for disaster risk reduction: A case study of the eastern Free State, South Africa

**DOI:** 10.4102/jamba.v10i1.400

**Published:** 2018-03-27

**Authors:** Johannes A. Belle, Nacelle Collins, Andries Jordaan

**Affiliations:** 1 Disaster Management Training and Education Centre for Africa, University of the Free State, South Africa; 2 Free State Department of Environmental Affairs, Bloemfontein, South Africa

## Abstract

This article investigated the knowledge and practice of a nature-based solution to reduce disaster risks of drought, veld fires and floods using wetlands in the eastern Free State, South Africa. A mixed research method approach was used to collect primary data using three data collection tools, namely questionnaires, interviews and field observations. Ninety-five wetlands under communal and private ownership as well as a few in protected areas were sampled, with their users completing questionnaires. The study showed that communal wetlands were more degraded, while wetlands in protected areas and in private commercial farms were in a good ecological state. An extensive literature review reveals that healthy wetlands are effective buffers in reducing disaster risks such as drought, veld fires and floods which are recurrent in the study area. Therefore, through better land-use and management practices, backed by education and awareness, wetlands could be good instruments to mitigate recurrent natural hazards in the agriculturally dominated eastern Free State in South Africa.

## Introduction

The eastern Free State where this study was conducted is located in the Free State province, which is one of the nine provinces of the Republic of South Africa. The demarcation of the eastern Free State in this study did not follow any specific political or ecological boundary but was arbitrarily designed to closely follow the 28 ºE meridian and a vertical line that passed through the 500 mm – 700 mm isohyets; east of this line, rain-fed agriculture (the dominant activity in the Free State province) is feasible. The demarcated study area also separated the dry grassland in the west from the moist grassland in the east (Collins 2011; RSA DST 2010) (see Figure 1). The chosen study area was also large enough to include many wetland types in the province.

**FIGURE 1:**
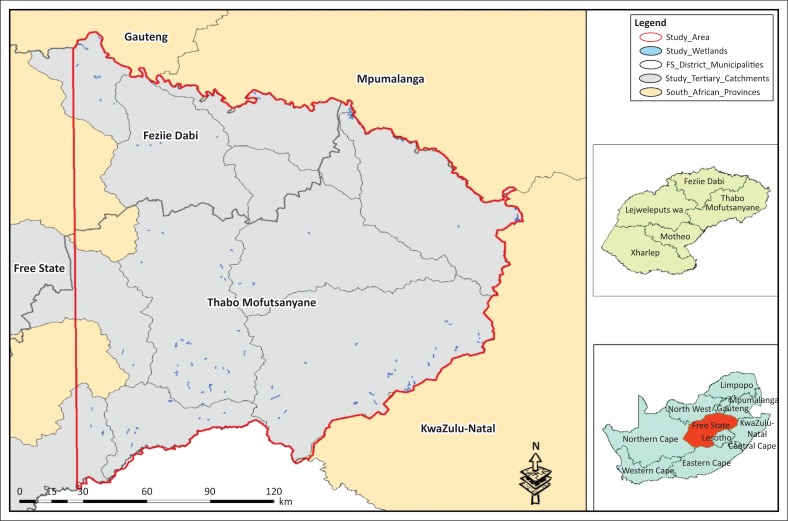
The eastern Free State.

### Definition of wetlands

It is not easy to define a wetland because they are of different types, and delineating wetland boundaries is problematic (Barbier, Acreman & Knowler 1997). Wetlands cover a wide range of habitats from freshwater marshes and wet meadows to estuarine mangroves and swamps (Kotze 2008). Known as *mokhoabo* in Sesotho, *umgxobhozo* in isiXhosa, *vlei* in Afrikaans and *wetlands* in English, wetlands have different names in South Africa. The *South Africa National Water Act* (1998) defines a wetland as:

Land which is transitional between terrestrial and aquatic systems where the water table is usually at or near the surface, or land that is periodically covered by shallow water and which in normal circumstances support or would support vegetation that is typically adapted to saturated soils. (p. 9)

This is a descriptive definition which uses hydrology, soil and vegetation to define a wetland. The above definition differs slightly from that of the Ramsar Convention on Wetlands definition whereby dams, rivers and shallow marine areas are considered to be wetlands. Generally, wetlands are transitional areas between aquatic and terrestrial ecosystems; and water, soil and vegetation are often used to delineate a wetland (Ayoade 2004; Pennington & Cech 2010; Ramsar Convention Secretariat [RCS] 2010; Republic of South Africa 1998; Wetland International [WI] 2015). The definition adopted in this article is that of the *Republic of South Africa Water Act* (1998).

### Functions of wetlands

Wetlands provide a variety and valuable ecological services to the local communities and these services are normally grouped into provisioning, regulating, cultural and supporting services (Kotze 2008; Millennium Ecosystem Report [MA] 2005; RCS 2010; The Economics of Ecosystems and Biodiversity [TEEB] 2010). Some authors refer to wetlands as *the kidneys of the landscape* because of their functions in the hydrological and chemical cycle or as *biological supermarkets* because of the extensive food web and rich biodiversity that they support (Barbier et al. 1997; RCS 2010; Russi et al. 2013; TEEB 2010). Wetlands directly reduce disaster risks through the natural regulatory processes and indirectly by providing scope for local livelihoods and reducing poverty, which are documented causal factors of disasters (Coppola 2011; Renaud et al. 2016; Renaud, Sudmeier-Rieux & Estrella 2013; United Nations International Strategy for Disaster Reduction [UNISDR] 2015; Wisner et al. 2004). The specific role of wetlands as an ecosystem in reducing disaster risks, adapting to climate change and supporting sustainable development was highlighted directly and indirectly during three important international agreements signed in 2015. These agreements included the Sendai Framework for Disaster Reduction 2015–2030 (UNISDR 2015), the Paris Climate Change Agreement (UNFCCC 2015) and the Sustainable Development Goals (UNDP 2015).

Generally, healthy and well-managed wetlands reduce disaster risks by acting as natural buffers against multiple hazards (Dudley et al. 2015; Partnership on Environment and Disaster Risk Reduction and Centre for Natural Resources and Development [PEDRR & CNRD] 2013; RCS 2010; Renaud et al. 2013). Healthy wetlands also build local resilience against disasters by sustaining local livelihoods through the provision of important products like wild fruits, vegetables, fish and padi rice to the local population (Kotze 1997, 2008; MA 2005; PEDRR & CNRD 2013; RCS 2010). The regulatory role of wetlands such as climate regulations also helps to reduce the intensity and frequency of weather and climate-related hazards (Intergovernmental Panel on Climate Change [IPCC] 2014; UNISDR 2015; WI 2016).

Flood plains and valley-bottom wetlands attenuate flood water by dispersing the incoming water, breaking the energy of the water and slowing down the speed of movement of the water through the wetland (Collins 2006; RCS 2010; Renaud et al. 2016). In addition, flood plains, valley bottoms and even seep wetlands mitigate dry spells and drought by providing water and fodder for grazing (Kotze 2008). Peat wetlands are effective for carbon sequestration and thus reduce global warming and the associated climate-related disasters like storms (IPCC 2014; UNISDR 2015).

### Wetlands in the study area

**FIGURE 2:**
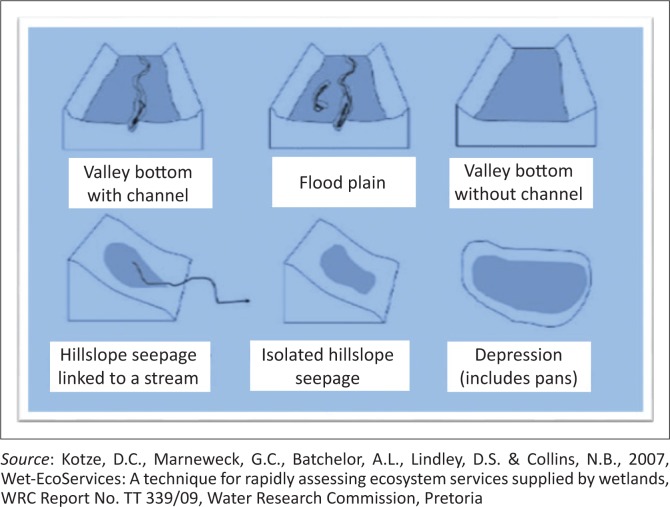
The hydro-geomorphic classification of wetlands.

According to the National Freshwater Ecosystem Priority Areas (NFEPA), the Free State has the highest number of wetlands in South Africa (South African National Biodiversity Institute [SANBI] 2010 in Collins 2011). There are about 54 000 natural wetlands in the Free State. These wetlands comprise valley-bottom units, flood plains, slopes and pans (Figure 2) (Collins 2006; Ollis et al. 2013). The dominant wetlands in the eastern Free State are channelled, valley-bottom wetlands and an estimated 2624 of such wetlands were identified in the study area (Collins 2011).

In this study, all but one of the 95 sampled wetlands were valley-bottom wetlands and were divided into private and communal wetlands whereby all wetlands under an identifiable ownership were considered to be private while those with collective ownership were classified as communal.

### Identified disaster risks facing wetlands in the study area

Disaster risk is a product of a hazard affecting a vulnerable community or system that lacks coping or adaptive capacities (Birkmann et al. 2013; Coppola 2011; Wisner et al. 2004). Many disaster risks that exist in the Free State province have impacts or potential impacts on wetlands. The Free State Provincial Disaster Management Plan identified the following hazards that pose disaster risk in the province: drought, floods, veld fires, structural fires, epidemics, extreme cold, heat waves, hail, windstorms, tornadoes, earthquakes, sinkholes, hazardous materials (Hazmats), transport accidents, seismic movements, dam failures, snow, mudslides and water contamination (FSPDMC 2007). Wetlands can play a great role in mitigating hazards, especially those associated with drought, floods and veld fires (Kotze et al. 2007; Renaud et al. 2016; WI 2016).

Thabo Mofutsanyane is the main district municipality in the eastern Free State. From the various presentations from the districts at the quarterly National Disaster Management Advisory Forums, it is clear that droughts and veld fires are among the top four recurrent risks in the eastern Free State, the other two being epidemics and floods (FSPDMC 2007). Therefore, having many wetlands in the study area where there is a recurrent risk of drought, floods and veld fires makes a pertinent study on how knowledge and careful management of these wetlands could reduce disaster risks in the eastern Free State.

## Methods

A multidisciplinary and mixed-method approach was used to collect primary data. Mitigating disaster risks requires the integration of knowledge from many spheres, which include the natural-, engineering- and social sciences (Birkmann et al. 2013; IPCC 2014; Takeuchi et al. 2014). The mixed-method approach made it easy to generate quantitative and qualitative data, and to incorporate a combination of post-positivism and interpretivism paradigms in the study (Bertram & Christiansen 2014; Creswell 2003, 2014; Okeke & Van Wyk 2015).

Understanding the risks and vulnerabilities of wetlands was crucial in this study. Any risk is a product of the hazard (H) and vulnerability (V) compared with the coping or adaptive capacity (C) of the community, structure or system (R = H × V/C) (UNISDR 2004; Wisner et al. 2004; Wisner, Gaillard & Kelman 2012). While the hazards were identified in this study (drought, floods and veld fires), there was a need to assess the vulnerability and adaptive capacities of the wetlands to determine their abilities to mitigate these hazards. In disaster management, risk is seen as the probability of the occurrence of a harmful event with negative consequences (UNISDR 2009). A hazard is seen as a dangerous phenomenon, substance, human activity or condition that can negatively affect humans, their social organisation or their environment, while vulnerability is the degree to which a society or system is susceptible to the impact of a hazard (UNISDR 2009). The coping or adaptive capacity is the inherent and organisational ability of a community or system to absorb and resist the impacts of hazards (Coppola 2011; UNISDR 2009). The ecological status of a wetland or how intact the wetland is as a system influences the vulnerability and the ability of the wetland to cope with natural hazards like floods, veld fires and drought.

Ten indicators were designed and used (adapted from Oberholster et al. 2014) to observe 21 wetlands in the study area to determine the ecological status of these wetlands. The 10 indicators included the wetland size, land-use type, hydroperiod, vegetation cover, alien species, pollution, sedimentation, grazing carrying capacity, activities within the wetland and bank stability and/or erosion. These indicators were not weighted, but their varying influences on wetlands was noted. The indicators were based on easily observable clues in a wetland even by a non-wetland specialist. Each indicator was scored from the best value of 5 to the worst value of 1 (see Appendix 1). A zero score was not allocated, because it could mean the indicator did not exist in the wetland at all. The total score was 50, which was later converted into a percentage and grouped into four ecological status categories: excellent = more than 75%; good = 65% – 75%; average = 50% – 64%; poor = less than 50%.

Three data collection tools were used, comprising questionnaires, interviews and field observations. A total of 176 valid questionnaires from 93 communal wetland users and 83 private wetland users were analysed. For the purpose of assigning responsibility and accountability in wetlands management to an identifiable individual, only two categories of wetland owners and/or users were applied. Where the owner of the wetland could be identified, such a wetland was classified as ‘private’. The private wetland users therefore included those on private commercial farms and government-owned wetlands (i.e. those located within conservation agencies like SANParks), to distinguish them from communally owned wetlands which were collectively owned without an identifiable manager. In total, 95 wetlands were sampled. Face to face and telephonic interviews were conducted with five wetland specialists, eight environmental and disaster management specialists and two environmental law specialists in the Free State province. Lastly, though field observations were carried out in most of the wetlands during the administration of the questionnaire, detailed observations were carried out on 21 randomly selected wetlands (7 communal wetlands, 11 privately owned wetlands and 3 protected wetlands). The communal wetlands were located in Monontsa, Bethlehem, Clarens, Heilbron, Petrus Steyn, Edenville and Frankfort, and the privately owned wetlands in Swineburne and Van Reenen’s Pass in the Harrismith area. The three protected wetlands included Seekoeivlei, Golden Gate and the Eskom wetland systems at Ingula Power Station. Furthermore, a pilot study was conducted in six wetlands: two in protected areas, two on communal land and two on private land. Three Master’s students, three PhD students and three senior researchers tested the questionnaire before the pilot study. These measures added validity and reliability to the data (De Vos et al. 2005; Polit & Hungler 1999; Saunders, Lewis & Thornhill 2000).

The SPSS version 23 was used to analyse the quantitative data, while the qualitative data were inductively analysed into dominant themes that emerged from the raw data (Creswell 2014; Maree 2007). The Kendall’s W Test was performed to explore what the private wetland owners perceive as the current and future major threats to their wetlands as part of the vulnerability assessment of wetlands in the area.

## Results

### Demographics of the respondents

The demographic data of communal wetland users showed that more men than women completed the questionnaire, that they were of middle age and that they were mostly unemployed or self-employed (Table 1).

**TABLE 1:** Summary of the demographic background of communal wetlands respondents.

Parameter	Frequency	Percentage of total respondents
**Gender**		
Female	35	37.6
Male	58	62.4
**Median age**	30–39	51.6
**Employment status**		
Unemployed	39	41.9
Self-employed	20	21.5
Employed	34	36.6
**Number of years using the wetland (more than 5 years)**	84	92.3
**Owner of the wetland**		
Government	38	40.9
Communally owned	34	36.6
Do not know	17	18.3

Most of the private wetland owners were male, with a median age of between 45 and 54 years. Many (77.1%) had used the wetland for more than five years, of which 60% had more than 10 years’ experience (Table 2). Most private wetland owners were commercial famers, whilst communal wetland users mostly practiced communal, small-scale grazing.

**TABLE 2:** Summary of the demographic background of private wetland owners.

Parameter	Frequency	Percentage of total respondents
**Gender**		
Female	16	19.3
Male	67	80.0
**Median age (years)**	45–54	54.2
**Mean age (years)**	51.98	-
**Modal age (years)**	55–64	31.3
**Education**		
Primary	7	8.4
Matric	17	20.5
Undergraduate	30	36.1
Postgraduate	29	34.9
**Number of years using the wetland**		
More than 5 years but less than 10 years	64	77.1
More than 10 years	42	50.6

### Wetlands threats, risks and vulnerability

Communal wetland respondents indicated that flood and veld fires affect them more, while private wetland owners felt that they were more affected by floods than drought as they use their wetlands mostly for commercial agriculture (Table 3). It should be noted that primary data were collected during the onset of the 2014–2016 prolonged drought that affected the whole of southern Africa including the eastern Free State. Possibly, the same data collected from the same respondents after the drought could see drought at the top of their risk profile, given the huge impacts that the 2014–2016 drought had on the area.

**TABLE 3:** Common risks experienced in communal and private wetlands.

Hazard	Responses	Communal (*n* = 93)		Private (*n* = 83)	
		Frequency	Percentage	Frequency	Percentage
Flood	No	31	33.3	19	22.9
	Yes	62	66.7	63	75.6
Drought	No	79	84.9	47	56.6
	Yes	14	15.1	36	43.3
Fire	No	66	71.0	52	63.0
	Yes	27	29.0	31	37.0

Besides the above mentioned threats, private wetlands owners also perceived a lack of awareness on wetland benefits to be a major threat to their wetland (Table 4).

**TABLE 4:** Perceived wetland threats to private owners: Kendall’s W Test (ranks).

Threat	Mean rank
Invasive alien species	6.19
Overgrazing	7.64^c^
Uncontrolled fire	8.81^b^
Lack of awareness about wetland benefits	8.94^a^
Soil erosion	6.96
Sedimentation	7.23
Pollution	6.12
Climate variability	5.65
Change in water regime	6.45
Conversion to other uses	5.87
Lack of human management capacity	6.70
Lack of material resources to manage	7.14
Upper catchment management activities	7.28^d^

Test statistics: *N* = 83; Kendall’s *W*^a^ = 0.93; Chi-square = 92.91; *df* = 12; Asymp. Sig. = 0.000.

a,b,c,d , indicates threat parameters ordered per the mean rank.

### Inappropriate land-use practices that lead to wetland degradation

The following activities were reported by respondents as poor wetland management practices that led to wetland degradation:

- Overgrazing. This practice stemmed from overstocking livestock and game as well as the use of the wrong game species. Overgrazing was generally observed during the dry winter season.
- Poor fire management planning as well as uncontrolled runaway fires.
- The introduction of both invasive and alien species in and around the wetland.
- The drainage of wetlands for agricultural practices, human settlement and road construction.
- The construction of dams for irrigation and electricity production.
- The pollution of wetlands from heavy agricultural chemicals and poor waste disposal.
- The uncontrolled harvesting of wetland vegetation, herbs and medicinal plants.
- Poor grazing practices such as grazing the permanently wet areas of the wetlands, which causes animal trampling.

### Ecological status of case study wetlands

The ecological status of a wetland was used as a proxy to assess its level of vulnerability to imminent hazards and consequently its ability to mitigate those hazards. Respondents were requested to score the current state of their wetlands against the key wetland parameters of vegetation, water and soil (Table 5). The results showed that 67.5% of private wetlands owners reported that their wetland vegetation was either in a good or very good ecological state, 63.9% said the hydrology in their wetland was either good or very good while 60.3% reported that the soil was either good or very good. As indicated later, the hydrology and vegetation of the communal wetlands were in a poor state. This information supported what was observed in the field.

**TABLE 5:** The ecological status of the key components of wetlands by private wetland owners.

Ecological status	Poor (1)		Fair (2)		Good (3)		Very good (4)		Mean score
	*n*	%	*n*	%	*n*	%	*n*	%	
Vegetation	6	7.2	21	25.3	43	51.8	13	15.7	2.76
Water	13	15.7	17	20.5	35	42.2	18	21.7	2.70
Soil	9	10.8	24	28.9	35	42.2	15	18.1	2.67

The ecological status derived from the 10 indicators is summarised in Table 6. All the communal wetlands except one had a poor ecological status. In contrast, privately owned wetlands had either an excellent or good ecological status, with only one wetland having an average ecological status.

**TABLE 6:** Ecological status of valley-bottom wetlands from field observation.

Ownership	Wetland group	Number	WL ID	Score/50	% score	Ecological Status
Communal	-	1	Monontsha	20	40	Poor
		2	Bethlehem	18	36	Poor
		3	Helbron	24	48	Poor
		4	Frankfort	23	46	Poor
		5	Petrus Steyn	24	48	Poor
		6	Edenville	22	44	Poor
		7	Clarens	27	54	Average
Private	Protected government	8	Seekoevlei	45	90	Excellent
		9	Ingula	40	80	Excellent
	Protected SANParks	10	Golden Gate	39	78	Excellent
	commercial farms	11	SB1	36	72	Good
		12	SB2	36	72	Good
		13	SB3	34	68	Good
		14	SB4	34	68	Good
		15	VR1	35	70	Good
		16	VR2	36	72	Good
		17	VR3	33	66	Good
		18	VR4	34	68	Good
		19	FB1	41	82	Good
		20	RT1	33	66	Good
		21	QQ1	31	62	Average

The results from field observations tally with the interview results from five wetland specialists. Based on their past experiences, all specialists reported that protected wetlands at Golden Gate, Mamel and Ingula were in a very good condition apart from a few head-cut erosions. They also reported that most wetlands on private commercial farms were in a good state. Those identified as having problems were being rehabilitated by the Working for Wetlands Programme. Communal wetlands were generally in a poor state despite efforts to rehabilitate some of these areas. The main problems that were reported in communal wetlands were open, uncontrolled grazing and other commercial activities like sand excavation within the wetlands. The conversion of the Dihlabeng wetland in Bethlehem into a mall was also cited.

### Managing wetlands for disaster risk reduction

The communal respondents responded negatively when asked whether wetlands help them to reduce the common hazards in the area (veld fires, drought and floods). Their responses are summarised in Table 7. However, private wetland owners agreed that they manage their wetlands in order to reduce the common disaster risks of drought, veld fires and floods as indicated in Table 8.

**TABLE 7:** The capability of wetlands to reduce the impacts of flood, drought and fire in communal wetlands.

Hazard	Response	Frequency	Percentage	Cumulative percentage
**Drought**				
Valid	No	64	68.8	68.8
	Yes	29	31.2	100.0
	**Total**	**93**	**100.0**	**-**
**Fire**				
Valid	No	65	69.9	69.9
	Yes	28	30.1	100.0
	**Total**	**93**	**100**	**-**
**Flood**				
Valid	Yes	74	79.6	79.6
	No	19	20.4	100.0
	**Total**	**93**	**100.0**	**-**

**TABLE 8:** Management of private wetlands to reduce disaster risks.

Hazard	Response	Frequency	Percentage	Cumulative percentage
**Drought**				
Valid	Agree	58	69.9	69.9
	Disagree	23	27.7	97.6
	Undecided	2	2.4	100.0
**Fire**				
Valid	Agree	54	65.1	65.1
	Disagree	27	32.5	97.6
	Undecided	2	2.4	100.0
**Flood**				
Valid	Agree	50	60.3	60.3
	Disagree	31	37.3	97.6
	Undecided	2	2.4	100.0
**Climate change**				
Valid	Agree	38	45.8	45.8
	Disagree	42	50.6	96.4
	Undecided	3	3.6	100.0

In private wetlands, 69.9% had no wetland management plans, while 12% had plans that were seldom used and revised. A high percentage of these wetlands (85.5%) had a limited protection status or none at all. Another 75.9% reported that they did not know the threats facing their wetland, and therefore could not address the threats or insufficiently addressed them. Another 85.5% either had no mechanisms in place to control inappropriate land-use activities, or the mechanisms were ineffectively implemented. What was noted from these responses was that there was no comprehensive and holistic knowledge of wetland threats in the area. Besides the threats of drought, floods and veld fires, there are other stressors that affect wetlands in varying degrees in the area – drainage and land-use conversion of wetlands. The conversion of the Dihlabeng wetland in Bethlehem into a mall, climate change, ineffective implementation of policies related to wetlands, ignorance of the functions and values of wetlands and so on were either observed or reported during interviews.

### Suggestions to better manage wetlands

Table 9 summarises respondents’ suggestions on ways that wetlands could be utilised and better maintained. Providing education and training as well as more effective laws and policies ranked the highest.

**TABLE 9:** Suggestions on how to better manage wetlands in the area from both private and communal users.

Suggestions	Frequency	Rank
Provide education and training on wetlands	12	1
Effective wetland laws and policies	7	2
Provide dumping sites, and rubbish cans, and control pollution	5	3
Relocate the settlers and provide better land	5	3
Build bridges and other forms of flood control	4	4
Provide fodder, especially in winter	3	5
Create jobs for the local people	3	5
Provide water-saving devices	3	5
Fence round the wetlands	2	6

### Dominant wetland uses in the area

The field observations showed that most of the wetlands in the study area were used for grazing. For example, all communal wetlands were used for small-scale grazing. A few wetlands were cultivated – mainly for maize, beans and sunflowers – and only two wetlands were used entirely for conservation. This information is summarised in Table 10.

**TABLE 10:** Dominant land use within the sampled wetlands.

Activity	Frequency	Percentage
Grazing	76	80.0
Crop cultivation	8	8.4
Mixed (crop and grazing)	5	5.2
Conservation and grazing	4	4.2
Conservation	2	2.2

**Total**	**95**	**100.0**

## Discussion

### Wetland risks

Respondents from communal wetlands indicated that they were more vulnerable to the risk of floods than veld fires and drought. This can be explained by many factors. First, the communal wetlands that were sampled were channelled, valley-bottom wetlands, which easily collect and channel rainfall in the catchment. Second, unlike flood plain wetlands, valley-bottom wetlands are less efficient in attenuating flood waters and mitigating the risk of floods (Kotze 2008; RCS 2010). Third, there are many informal settlements within and around the communal wetlands with a high risk of floods, even with the slightest bank overflow. Last, surface-concreting from the informal settlements, road constructions and draining of wetlands for various other reasons increase the risk of floods around communal wetlands as observed in Heilbron, Monosta and Petrus Steyn. Here, head cut in the wetlands could easily be attributed to settlement and road concreting, which increased the volume and energy of the flow of water entering the wetlands. These wetlands, including those in private holdings, however, play a better mitigation role against the risk of fires and drought, given the continuous presence of water or moisture. This is valid even in winter as the study area falls within the summer rainfall zone of South Africa. The presence of water or moisture in wetlands even during dry spells and droughts could be used to motivate for wetland conservation. The risks of climate change, overgrazing and uncontrolled fires were also reported. It should be noted, however, that data were collected before the 2014–2016 drought (the worst drought in 50 years in the area) that could possibly have altered the responses.

Foremost in the ranking of perceived threats to private wetlands was the lack of awareness of wetland benefits, followed by uncontrolled fire and then overgrazing. The Kendall’s W Test confirmed the perception that there was an urgent need for education and training on wetland management. The test statistic for the ranking of the threats revealed that about 93% of the private wetland owners agreed to a ranking order as provided in Table 4. The Chi-square statistic of 92.91 was highly significant at 1% level, suggesting that the ranking was valid and efficiently estimated. This further showed that the individual threats identified in the study jointly and significantly explain the actual threats to the eastern Free State wetlands.

From the field observations, six out of seven communal wetlands were in a poor state, with only one in an average ecological state. All the wetlands in protected areas were in an excellent ecological state, with one of them (Seekoevlei) being a Ramsar site. The Ingula wetland (one of the protected wetlands) could eventually qualify for a Ramsar site designation, given its present status and ecological functions. Wetlands found on private commercial farms were clustered around a good ecological status. One of them was in excellent ecological health, and this wetland is also a heritage site. Most protected wetlands were therefore in a very good to excellent ecological status; those on private land were in an average to good ecological status; and those on communal land mostly had a poor ecological status (Table 6). This state of affairs could be linked to many factors, ranging from ignorance, land title and private interest to management style and non-existence or weak implementation of wetland laws or environment-related laws. While monitoring is required to maintain the excellent ecological state of protected wetlands, it would be good management practice to improve the status of wetlands on private commercial farms from good to excellent. Communal wetlands, however, need the most careful planning.

### Wetlands management for disaster risk reduction

Wetlands can be managed to reduce the impact of disaster risks as well as to adapt to climate change. This is popularly referred to as the ecosystem-based disaster risk reduction and climate change adaptation (Eco-DRR/CCA) approach, (PEDRR & CNRD 2013; Renaud et al. 2013; UNEP 2009).

Most of the communal wetland users do not perceive wetlands as having any mitigation effects on the common hazards of drought and veld fires in the area. They would therefore not possibly manage these wetlands for disaster risk reduction. This situation again demonstrates the lack of awareness and education among the communal wetland users regarding the potential of wetlands to reduce many of the disaster risks in the area. This lack of awareness may contribute to the degradation of most communal wetlands. One of the most reported problems by the communal wetlands users who completed the questionnaire was uncontrolled fire; yet, these users do not see wetlands as a possible fire mitigation factor, even by the sheer presence of water in some parts of the wetlands in winter. Wetlands can be used as effective fire breaks (FSPDMC 2007). The private wetland owners agreed that they manage their wetlands in order to reduce the common disaster risks of drought, veld fires and floods. Most private wetland users try to avoid overgrazing so that their wetlands continue to provide fodder even during dry spells and droughts. They also use structural measures like gabions to break the force of water entering their wetlands and get an even spread of water in the wetland. Some use wetlands as fire breaks to mitigate the impact of runaway fires. This holistic view was contrary to what was reported and observed in the communal wetlands.

### Suggestion to better manage wetlands

Top on the list of suggestions (based on frequency of suggestions) from both private and communal wetland users was the need to provide education and training on the importance, conservation, protection and wise use of wetlands (Table 9). This was followed by formulating and implementing stringent laws on wetlands. The latter should possibly be the joint effort of the government and the local municipalities. Third on the list was the plea that dumping sites, rubbish bins and other forms of pollution control be put in place. This is important as communal wetlands were observed to be heavily polluted, especially from domestic waste. All the communal wetlands that were sampled were surrounded by informal settlements that generate domestic waste. There were suggestions that the government should relocate the people who settle in wetlands and provide better livelihoods for them. The land issue in the study area, as in the rest of South Africa, is imbalanced and complicated, having its root causes in the discriminatory era of apartheid. The provision of fodder, especially in winter, was also mentioned, as was job creation and the provision of water-saving devices like water tanks, which could ease pressure on wetlands that were used for water harvesting. Fencing the wetlands can be very expensive and was the least important on the list of suggestions.

The common ownership with no control over communal wetlands makes the management planning of wetlands almost non-existent in the study area. There were no management plans, written or unwritten, for communal wetlands and there was no observed control of illegal activities such as pollution. For example, at the Monontsha wetland, a channel was constructed to direct waste from a pig sty into the wetland, causing pollution to downstream users. Private commercial farmers reported that they had management plans for their wetlands although these plans were not documented and regularly revised as opposed to those in protected areas.

With no adequate education, awareness and training on wetlands management, the absence of management plans in most wetlands (no specific wetland policy to guide wetland management in the study area), points to the fact that the fate of the majority of these wetlands depends on the ingenuity, guess work and experience of the individual users. Better management plans and processes were observed in the three protected and conserved wetlands that were included in this study (Seekoeivlei, Golden Gate and the Eskom wetland systems at Ingula Power Station). The Working for Wetlands Programme has, however, been rehabilitating many wetlands in communal and private commercial farms in the area, but this approach is too reactive and spontaneous.

## Conclusion and recommendations

Communal wetlands are in a very poor ecological state as opposed to protected wetlands and those on private commercial farms in the eastern Free State. The risk of veld fire and drought are high in the study area, and this has serious negative impacts on agriculture and grazing, which are the dominant activities in the Free State province. Grazing is the dominant economic activity in the sampled wetlands. Well-managed wetlands can effectively reduce the risk of veld fires, floods and drought, as observed in the field and supported by the literature review. This is the case with wetlands in protected areas and on private commercial farms in the study area. On the contrary, degraded wetlands lack the capacity to mitigate risks, as observed in communal wetlands. Education and awareness on the role of wetlands in reducing recurrent disaster risks in the area is crucial, especially among the communal wetlands users. The current climate variability adds to the need for education on proper wetland management, awareness and the development of holistic wetland management plans that should constantly be revised and carefully implemented to accommodate the changing external environment.

Proper training on the use and management of wetlands is also vital. The University of the Free State (UFS) and Central University of Technology (CUT), the leading tertiary institutions in the area, could design courses on wetlands management, while the Working for Wetlands Programme could offer skills enhancement courses to the local communities. The Mondi Wetland Programme (MWP) and the Endangered Wildlife Trust (EWT), which are prominent non-governmental organisations (NGOs) with an important role to play in wetlands management in South Africa, are not prominent in the study areas. They could also assist in capacity building in the area regarding wetlands issues.

It is important to rehabilitate the degraded communal wetlands as well as monitor the ecological status of wetlands on protected and private farms. This can be performed by the Working for Wetlands Programme and supported by the Working for Water Programme, which has the assigned responsibility from government of the clearing of invasive species that may include those in wetlands. These programmes should be more capacitated with both financial and human resources so that they can function effectively.

Wetlands could be a cost-effective, community-driven, bottom-top approach in mitigating the recurrent risks of drought, veld fires and floods in the eastern Free State if wetlands are properly managed. This ecosystem-based approach to reduce disaster risk and adapt to climate change has received and continues to receive much international attention in recent years.

## Figures and Tables

**FIGURE 1-A1:**
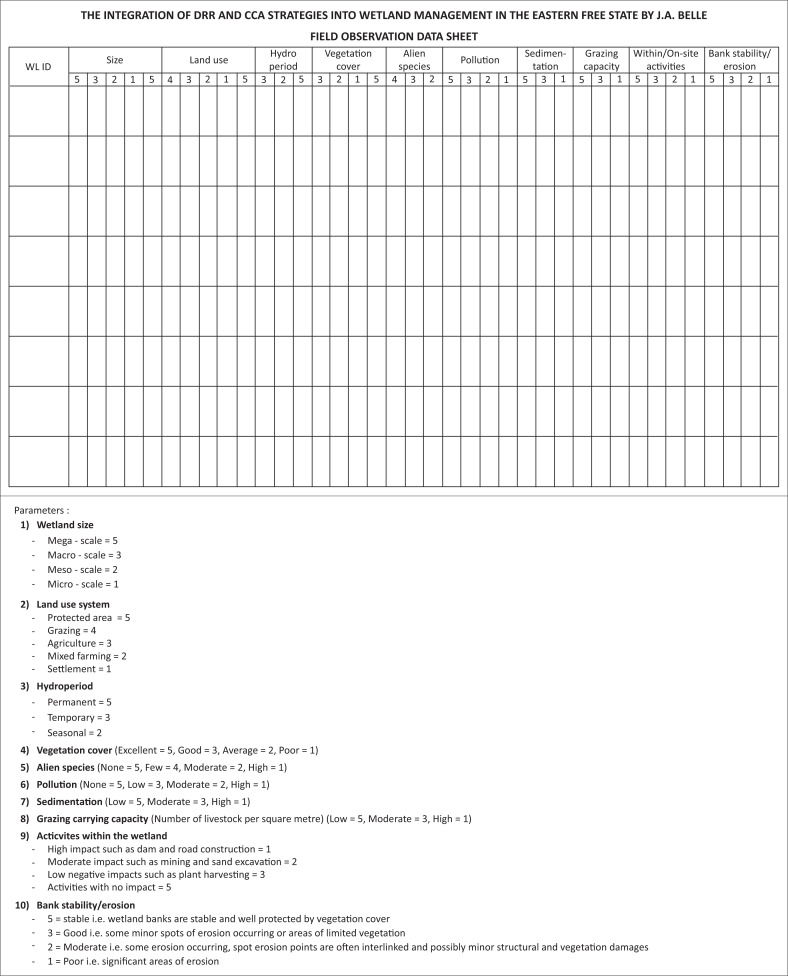
Field observation data sheet.
